# The role of nurses and midwives in polio eradication and measles control activities: a survey in Sudan and Zambia

**DOI:** 10.1186/1478-4491-7-78

**Published:** 2009-09-08

**Authors:** Annette Mwansa Nkowane, Liliane Boualam, Salah Haithami, El Tayeb Ahmed El Sayed, Helen Mutambo

**Affiliations:** 1 Department of Human Resources for Health, World Health Organization, Geneva, Switzerland; 2 Polio Eradication Initiative, World Health Organization, Geneva, Switzerland; 3 World Health Organization Country Office, Khartoum, Sudan; 4 Federal Ministry of Health, Khartoum, Sudan; 5 World Health Organization Country Office, Lusaka, Zambia

## Abstract

**Background:**

Nurses and midwives are the key providers of nursing and midwifery services; in many countries, they form the major category of frontline workers who provide both preventive and curative services in the community. When the skills and experience of nursing and midwifery personnel are maximized, they can contribute significantly to positive health outcomes. We conducted a survey among nurses and midwives working at district level in Sudan and Zambia to determine their roles and functions in polio eradication and measles elimination programmes.

**Methods:**

Nurses and midwives practising in four selected districts in Sudan and in Zambia completed a self-administered questionnaire on their roles and responsibilities, their routine activities and their functions during supplementary immunization campaigns for polio and measles.

**Results:**

Nurses and midwives were found to play significant roles in implementing immunization programme activities. The level of responsibilities of nurses and midwives in their routine work related more to existing opportunities than to their job descriptions.

In Zambia, where nurses reported constraints in performing their tasks, the reasons cited were an increase in the burden of disease and the shortage of health personnel. Factors identified as key to improving work performance included written job descriptions, opportunities for staff and career development and opportunities to earn extra income through activities associated with their jobs.

Other non-monetary incentives mentioned included reliable transport, resources and logistics to support routine work in the district. However, in both countries, during supplementary immunization activities or mass campaigns for polio eradication and measles control, nurses and midwives took on more management responsibilities.

**Conclusion:**

This study shows that nurses and midwives play an important role in implementing immunization activities at the district level and that their roles can be maximized by creating opportunities that lead to their having more responsibilities in their work and in particular, their involvement in early phases of planning of priority health activities. This should be accompanied by written job descriptions, tasks and clear lines of authority as well as good supportive supervision. The lessons from supplementary immunization activities, where the roles of nurses and midwives are maximized, can be easily adopted to benefit the rest of the health services provided at district level.

## Background

Human resources are crucial to effective delivery of health services. Strategies across countries are, however, constrained by patchy evidence, limited planning tools and a scarcity of technical expertise [1].

Nursing and midwifery services are a subsystem of health services that are provided by a wide range of personnel. These services share common attributes such as: prevention of diseases; caring for, supporting and comforting clients; continuously assessing and monitoring health needs and responses to interventions; advocacy and education of clients and communities; and delivering and coordinating health services across the care spectrum [2]. Nurses and midwives are the key health care providers and constitute the largest proportion of the health workforce globally. In most countries, they are the first, and often the only, point of contact for patients, and in many rural areas they often provide as much as 80% of required health care services [3].

Barriers to the delivery of primary health care interventions include lack of prescribing authority, lack of support from physicians, reimbursement difficulties and lack of public awareness [4]. Recent global initiatives such as polio eradication and measles elimination have led to the recognition that roles of nurses and midwives can be maximized and that they form a key human resource in activities that require mass community mobilization and involvement.

These lessons can be used to improve the delivery of other health services in many resource-constrained settings. This paper reports on a survey conducted in Sudan and Zambia in 2007, which looked at the roles of nurses and midwives in the expanded programme on immunization.

### Study objectives

The objectives of this study were to document the roles, functions and contribution of nurses and midwives in the delivery of routine immunization services and supplementary immunization activities for disease prevention, control or eradication and to identify the best practices that can be adopted for use in other priority health programmes.

### Study justification

One of the key strategies for addressing the current human resources crises in many countries is to maximize the roles of existing human resources for health. For nurses and midwives, who are core providers of health care services, changes in how their work is structured and in their perception of themselves can enable them to improve their contribution to the health system. Facilitating nurses to function at this level is necessary for the process of empowerment [5].

Given the pace of change in health systems across the world, the need to develop nurses and midwives who value creativity and innovation and enjoy the challenge of constant change has never been greater. Today's nurses need to be comfortable with reorganization of their roles and work patterns and to regard problem solving rather than problem identification as essentially the norm of operations [6].

## Methods

### Study sites

Two countries, Sudan and Zambia, participated in the study. These countries were selected because they had programmes for routine immunization for children (Expanded Programme on Immunization); had conducted supplementary immunization campaigns for polio eradication in the previous three years; had conducted supplementary immunization campaigns for measles in the last three to four years; and had an established surveillance system for vaccine-preventable diseases. In each participating country, a purposeful convenient sample of four districts was selected; in each district, four health facilities (two urban and two rural) were selected to participate in the survey.

### Study participants

In each district, two groups of health personnel were invited to participate in the survey. These were (1) the district EPI programme manager (or the equivalent person with overall responsibility for EPI and supplementary immunization activities (SIAs) for polio and measles) and (2) all nurses and midwives working on immunization/child health activities at health facilities in the participating district. Participation in the survey was voluntary and the study was conducted in August and September 2007.

### Study variables, data collection and data analysis

Structured, self-administered questionnaires were completed by the study participants. Both closed and open-ended questions were used. The information from the two groups of participants of the surveys fell into the following broad categories: (1) general information on EPI and supplementary vaccination activities in the district; (2) overall numbers and categories of health workers in the district; (3) the role of nurses and midwives in EPI and supplementary vaccination activities for polio and measles; (4) perceptions of nurses and midwives on how their role can be maximized or enhanced in EPI, supplementary vaccination activities and other priority health issues at district level; and (5) best practices and lessons learnt from EPI and supplementary immunization activities for disease eradication or control. The findings from the survey were descriptively summarized.

### Limitations of the study

The study is descriptive in nature and although comparisons can be made between the two countries that participated, standardized statistical testing and weighting of results could not be applied.

## Results

### Survey of district EPI managers

Eight district EPI managers, one from each district, participated in the survey. All the selected districts had conducted SIAs for polio eradication and measles in 2006 and 2007. The district managers in Zambia were nurse-midwives, while in Sudan they included nurses, midwives, medical doctors and environmental health officers. In both countries, the managers had been involved in EPI programme activities for an average of five years.

All the districts had functioning routine EPI programmes and the vaccine delivery strategies included fixed sites (where vaccines are provided at the health facility), outreach (where the health workers provide vaccine in the community at regular intervals), mobile delivery (where health workers move from place to place providing vaccinations) and campaigns. All districts had a DTP3 coverage (an indicator for routine immunization services) in the last two years of more than 75%, and in all the supplementary vaccination activities for both polio and measles, coverage achieved was more than 90% of the target population.

In both countries, the district managers were involved in all aspects of immunization programmes, including SIAs. These included planning, implementation, supervision of staff and evaluation of programme activities. In Sudan, the district managers reported that only 50% of the nurses were involved in all the seven functions during SIAs (management, planning, advocacy, social mobilization, training, implementation, supervision, monitoring and evaluation) and the same proportion was involved in AFP surveillance. On the other hand, in Zambia, nurses and midwives were involved in the full range of functions for SIAs and AFP surveillance.

### Survey of nurses and midwives working at health facilities

#### Routine tasks and service conditions of nurses and midwives

Forty-eight nurses and midwives participated in the survey. Twenty-six were from Sudan and 22 from Zambia. The proportion of midwives was 30.1% and 68.2%, respectively. The responses of the participants are presented in Table 1. A substantial proportion (38.5%) in Sudan and the majority in Zambia (63.6%) were aware of their written job descriptions. A higher proportion (92.3%) in Sudan, compared with 68.2% in Zambia, reported that the tasks they performed were consistent with their written or perceived job descriptions.

**Table 1:** Profile of respondents, routine tasks and service conditions

	**Variable**	**Sudan (n = 26)**		**Zambia (n = 22)**	
		**Number**	**%**	**Number**	**%**

1	Professional category				
	• Nurse/Midwife	8	30.1	15	68.2
	• Nurse	18	69.2	7	31.8

2	Respondent aware of written job description	10	38.5	14	63.6

3	Task consistent with job description	24	92.3	15	68.2

4	Tasks performed in a professional manner	21	80.8	8	36.4

5	Tasks match skills	21	80.8	8	36.4

6	Tasks take up too much time	13	50.0	17	77.3

7	Tasks delegated to others	2	7.7	18	81.8

8	Supervision:				
	• Regularly	20	76.9	16	72.7
	• Last 3 months	20	76.9	7	31.8

9	Adequacy of supplies	9	34.6	4	18.2

10	Transport and logistics considered adequate	6	23.1	2	9.1

11	Salaries and benefits considered adequate	6	23.1	2	9.1

12	In-service training provided to staff	24	92.3	14	63.6

13	Career advancement prospects available	19	73.1	7	31.8

Whereas 80.8% of the nurses in Sudan reported that the tasks in their routine work were performed in a professional manner and matched their skills, in Zambia this proportion was only 36.4%. The reason most commonly cited by the nurses in Zambia was that they spent more time dealing with diseases related to HIV infection, including HIV counselling - including prevention of mother to child transmission - referral of patients and administrative tasks. Furthermore, there was more delegation of tasks to other personnel in Zambia.

Supervision in both countries was reported as regular: Sudan (76.9%), Zambia (72.7%). Whereas all respondents in Sudan had received supervision in the three months prior to the study, the proportion in Zambia was only 31.8%. As regards supplies, logistics and salaries, including benefits, these were considered adequate: 34.6%, 23.1%, 23.1%, respectively, in Sudan, but by a much lower proportion in Zambia (18.2%, 9.1%, 9.1%). Lastly, in Sudan, 92.3% reported they had regular in-service training and 73.1% said they had career advancement prospects in their jobs. In Zambia, the proportions were lower (63.6% and 31.8%).

#### Factors perceived as improving work performance

Factors that were considered important to improving work performance are listed in Table 2. In Sudan, these were raising salaries (69.2%) and transport (57.7%). Other factors named by 34.6% respondents were training, good working environment and security and written job descriptions. In Zambia, the two factors cited most were transport (40.9%) and more staff (22.7%), whereas factors such as salaries including benefits and good working environment were named by only 18.2% of the respondents. Reported job-related incentives are shown in Table 3. Overall, bonuses, job-related reimbursements and official transport were cited by 42.3%, 30.1% and 30.1%, respectively, by the nurses in Sudan, while in Zambia, overtime (50.0%), bonuses (22.7%) and accommodation (18.2%) were cited most.

**Table 2:** Factors perceived to improve work performance

	**Factor**	**Sudan (n = 26)**		**Zambia (n = 22)**	
		**Number**	**(%)**	**Number**	**(%)**

**1**	**Better supplies/logistics**	1	3.8	2	9.1

**2**	**Training**	9	34.6	3	13.6

**3**	**Salary increase**	18	69.2	4	18.2

**4**	**Good environment/** **Security**	9	34.6	4	18.2

**5**	**Written job description**	9	34.6	-	-

**6**	**Transport**	15	57.7	9	40.9

**7**	**More incentives**	4	15.4	4	18.2

**8**	**More staff**	-	-	5	22.7

**Table 3:** Reported job-related incentives

	**Income source**	**Sudan (n = 26)**		**Zambia (n = 22)**	
		**Number**	**(%)**	**Number**	**(%)**

1	Bonuses	11	42.3	5	22.7

2	Job related reimbursements	8	30.1	-	-

3	Official transport	8	30.1	2	9.1

4	Accommodation provided	1	3.8	4	18.2

5	Hardship allowance	2	7.7	3	13.6

6	Overtime	-	-	11	50.0

#### Nurse-midwives' involvement in activities related to SIAs for polio and measles

##### Roles of nurses and midwives in SIAs for polio and measles

The involvement of the nurses and midwives in activities related to polio and measles SIAs is presented in Table 4. In general, the levels of involvement in some of the tasks differed between the two countries. In Sudan, management and leadership functions were reported by a small proportion for both polio and measles SIAs (23.1%). In Zambia, this was much higher (90.9% and 72.7% respectively). This finding is related to the fact that most nurses working at the health facility level in Zambia served in the capacity of district managers of health programmes. The finding that the nurses in Sudan were less involved in planning of activities (15.2% in polio and in measles) compared to 72.7% for polio and 90.9% for measles in Zambia, is also a reflection of the management functions.

**Table 4:** Roles of nurses and midwives in polio and measles SIAs

		**Sudan (n = 26)**		**Zambia (n = 22)**	
	**Variable**	**Polio SIAs**	**Measles SIAs**	**Polio SIAs**	**Measles SIAs**

**1**	Management/leadership	6 (23.1%)	6 (23.1%)	20 (90.9%)	16 (72.7%)

**2**	Planning campaigns activities	4 (15.2%)	4 (15.2%)	16 (72.7%)	20 (90.9%)

**3**	Advocacy/co-ordination	19 (73.1%)	19 (73.1%0)	19 (86.4%)	19 (86.4%)

**4**	Social Mobilization	21 (80.8%)	21 (80.8%)	19 (86.4%)	20 (90.9%)

**5**	Training	18 (69.2%)	17 (65.4%)	20 (90.9%)	20 (90.9%)

**6**	Implementation	25 (96.2%)	26 (100.0%)	20 (90.9%)	20 (90.9%)

**7**	Supervision of staff	25 (96.2%)	26 (100.0%)	20 (90.9%)	20 (90.9%)

**8**	Monitoring & Evaluation	3 (11.5%)	3 (11.5%)	21 (95.5%)	22 (100.0%)

On the other hand, advocacy, social mobilization, training implementation and supervision were reported in much higher proportions by nurses from both countries. Monitoring and evaluation for polio and measles SIAs were much lower in Sudan (11.5%, 11.5%) compared to 95.5% and 100.0% in Zambia.

##### Factors perceived as good for improving work performance during polio and measles SIAs

Table 5 lists factors and incentives cited by the respondents as conducive to good performance during SIAs for polio and measles. In Sudan, free, good-quality transport (84.6%), increased monetary incentives (73.1%), supervision during SIAs (69.2%), social mobilization (65.4%), good planning (61.5%), training (61.5%) and community participation (42.3% were the incentives most commonly cited. In Zambia the incentives most cited by the nurses were: provision of meals (86.4%); protective materials when giving injections (86.4%); free, good-quality transport (81.8%); adequate supplies (77.3%); social mobilization (63.6%); more qualified staff (59.1%); good planning (40.9%); and increased monetary incentives (40.9%).

**Table 5:** Factors and incentives for good performance during SIAs for polio and measles

	**Incentive/factor**	**Sudan (n = 26)**		**Zambia (n = 22)**	
		**Number**	**(%)**	**Number**	**(%)**

1	Free good quality transport	22	84.6	18	81.8

2	Provision of meals during campaigns	-	-	19	86.4

3	Increased monetary incentives during campaign	19	73.1	9	40.9

4	Training	17	65.4	1	4.5

5	Protective material	-	-	19	86.4

6	Supervision during SIAs	18	69.2	2	9.1

7	Community leader involvement	8	30.1	2	9.1

8	Social mobilization	17	65.4	14	63.6

9	Good planning	16	61.5	9	40.9

10	Training	16	61.5	8	36.4

11	Adequate supplies	1	3.8	17	77.3

12	More qualified staff	-	-	13	59.1

13	Community participation	11	42.3	1	4.5

##### Barriers to good performance during SIAs for polio and measles

The factors reported to be important barriers hindering good performance during immunization activities are listed in Table 6. In Sudan, the factors most commonly named were transport (50.0%), population illiteracy (34.6%), weak community involvement (34.6%), negative community perceptions (26.9%) and mobile populations (26.9%). In Zambia, the barriers were weak community involvement (59.1%), negative perceptions of community towards immunizations (54.6%), transport (31.8%), lack of supplies (31.8%) and wide distances to cover (27.3%)

**Table 6:** Barriers to good performance during SIAs for polio and measles

		**Sudan (n = 26)**		**Zambia (n = 22)**	
	**Factors**	**Number**	**(%)**	**Number**	**(%)**

**1**	**Mobile populations**	7	26.9	-	-

**2**	**Weather**	5	19.2	-	-

**3**	**Wide coverage area (distance)**	6	23.1	6	27.3

**4**	**Population illiteracy**	9	34.6	2	9.1

**5**	**Negative perceptions of community towards immunizations**	7	26.9	12	54.6

**6**	**Transport problems**	13	50.0	7	31.8

**7**	**Weak community involvement**	9	34.6	13	59.1

**8**	**Lack of supplies**	1	3.8	7	31.8

##### Lessons learnt from SIAs for polio and measles for improving work performance and optimizing their role

The main lessons learnt from supplementary vaccination campaigns for polio and measles control cited by the respondents that can be used to improve the work of a nurse or midwife were: understanding the community needs, including mapping (Sudan 65.4%); the importance of supervision (Sudan 53.8%); importance of good planning (Zambia 31.8%); and social mobilization and community participation (Zambia 22.7%). Salaries were cited by only 26.9% and 18.1% in Sudan and Zambia, respectively, as an important factor for improving work performance.

##### Best practices in SIAs for polio and measles

Nurses and midwives reported that they played a leading role in the implementation of supplementary immunization activities at the district level. When they served as district managers, they did all the planning and budgeting for activities. In addition, for the polio programme, nurses and midwives coordinated advocacy meetings at the district level and also organized sensitization meetings and workshops for the community. Other programme activities in which nurses played a significant role included social mobilization, training and serving as supervisors for other health workers in the district.

## Discussion

This case study on nurses and midwives working at district level and at health facilities in Sudan and Zambia provides valuable insight into the roles of nursing and midwifery personnel in a priority public health programme area. The finding that nurses and midwives in Zambia have more responsibilities than were observed in Sudan may be related to the organizational structure and nature of personnel in the district. In Zambia, the managers at the district are nurses and midwives.

Whatever the situation, however, in both countries nurses and midwives are able to perform all the functions for the programmes. The differences in responsibilities seem to be related more to opportunities available for nurses to take on management functions. This is reflected in the finding that during SIAs, except for management functions, the other functions are performed in similar proportions in both countries.

Delegation of responsibilities to others appears to be related to the management structure in place and possibly the level of training. In both countries, the nurses and midwives felt that the tasks they performed were consistent with their job description. However, the finding that only 38.5% in Sudan and 63.6% in Zambia reported being aware of their written job descriptions for their routine work was of concern. Studies show that job descriptions in addition to professional norms and codes of conduct influence personnel performance.

The *World health report *of 2006 suggested that there is need for proper matching of skills to the tasks at hand, supported by supervision. It appears, in the case of Zambia, that an increase in the burden of one disease, mainly HIV-related care, takes up much of the nursing personnel's time. Nurses were not performing the tasks related to their job descriptions and only 36.4% indicated that the tasks matched their skills and job descriptions.

Job descriptions that have clear objectives, responsibilities, authority and lines of accountability have been associated with improved attainment of work goals for all categories of health workers. Health worker motivation cannot be expected to be high in a situation where other tasks take up too much time.

The SIAs for polio eradication and measles control have provided an opportunity for nurses and midwives to take on more responsibilities. This in itself is a motivator and consequently leads to better job performance, especially when the tasks are clear and opportunities exist for performing management tasks during SIAs [7]. In order to perform the tasks well, working conditions are important. This study shows that while in Sudan, the work conditions were more or less favourable, in Zambia supplies, transport and salaries were considered inadequate and a constraint. In both countries, written job descriptions, opportunities for staff development and opportunities to earn extra income associated with their jobs were considered crucial to improving work performance.

The roles and functions of nurses and midwives during SIAs for polio and measles in both countries probably are the best example of how their roles can be maximized in programme implementation. Admittedly, SIAs for polio require the commitment of personnel for defined periods. However, the experience with participation in these activities and the roles played by nurses and midwives could be adopted in their routine work.

Important factors identified by the nurses and midwives that were crucial for improvement of performance are all the well-known factors such as good, effective supervision at all levels; planning of activities with community involvement; and proper training of persons involved in programme implementation. For supplementary activities, in contrast to routine activities, the barriers to implementation that were named tended to be non-human resources related (such as community involvement, adequate transport for operations and negative perceptions of the community to interventions related to immunizations.

The key lessons learnt from participation in supplementary vaccination activities for polio eradication and measles control include the importance of good planning, understanding community needs, good social mobilization and supervision during implementation. All throughout, good and reliable transport seems to be an important incentive or factor for improving performance.

## Conclusion

In summary, this study shows that nurses and midwives play an important role in implementation of routine and supplementary immunization activities at the district level. Their roles can be maximized by creating opportunities that lead to giving them more responsibilities, in particular their involvement in early phases of planning of priority health activities. This must, however, be accompanied by written job descriptions, tasks and clear lines of authority as well as good supervision. The lessons from supplementary immunization activities, where the roles of nurses and midwives are maximized, can be easily adopted to benefit the rest of the health services that are provided at district level.

## Competing interests

The authors declare that they have no competing interests.

## Authors' contributions

AMN conceptualized the project and wrote the study proposal, designed the questionnaires and initiated and participated in discussions, data analysis and writing of the article. LB participated in the conceptualization of the project, design of the study proposal, discussions, data analysis and review of the article. SH, ETAES and HM were involved in data collection, discussion and review of article. All authors have read and approved the final manuscript.
